# *In-silico* designing of acyclic nucleoside phosphonates and their anti-HIV potential

**DOI:** 10.1186/1471-2334-12-S1-P3

**Published:** 2012-05-04

**Authors:** Dipti Yadav, Anuradha Singh, Madhu Yadav, Ramendra K Singh

**Affiliations:** 1 Nucleic Acids Research Laboratory, Department of Chemistry, University of Allahabad, India

## Background

Reverse transcriptase, the viral enzyme, is a key target in the search for effective drugs useful for AIDS therapy and has critical roles in the life cycle of the human immunodeficiency virus type 1 (HIV-1), the causative agent of AIDS. HIV replication can be blocked by inhibition of the enzyme HIV RT.

## Method

The object of our study is to develop newer nucleoside phosphonate analogs bearing unsaturation and modifications in heterogenous bases and prediction of their anti-HIV potential. Designing is done keeping the Lipinski’s Rule of Five in focus. The diphosphates of compounds have been docked into the active site of wild type HIV-RT (PDB: ID 2B6A). The forcefield of the Chemistry at Harvard Macromolecular mechanics (CHARMm) was applied to 3D models of PD HIV RT-nevirapine complex and synthesized ligands. The energy function is based on separable internal coordinate terms and pair wise non-bond interaction terms.

## Result

Docking studies revealed that the diphosphates of acyclic phosphonates had good interactions with various amino acid residues present in the active site of HIV RT. The Ludi 3 score was found to be 718 and the corresponding K_d_ value was 0.06µM. This is in good agreement with the observed value of 0.05µM.

## Conclusion

On the basis of SAR studies, uridine phosphonate analogs are expected to be probable lead molecules against HIV-RT.

